# Comparative Utilization of Dead and Live Fungal Biomass for the Removal of Heavy Metal: A Concise Review

**DOI:** 10.1155/2021/5588111

**Published:** 2021-04-08

**Authors:** Abate Ayele, Setegn Haile, Digafe Alemu, M. Kamaraj

**Affiliations:** Department of Biotechnology, College of Biological and Chemical Engineering, Addis Ababa Science and Technology University, 16417 Addis Ababa, Ethiopia

## Abstract

Human and industrial activities produce and discharge wastes containing heavy metals into the water resources making them polluted, threatening human health and the ecosystem. Biosorption, the process of passive cation binding by dead or living biomass, represents a potentially cost-effective way of eliminating toxic heavy metals from industrial wastewater. The abilities of microorganisms to remove metal ions in solution have been extensively studied; in particular, live and dead fungi have been recognized as a promising class of low-cost adsorbents for the removal of heavy metal ions. The biosorption behavior of fungal biomass is getting attention due to its several advantages; hence, it needs to be explored further to take its maximum advantage on wastewater treatment. This review discusses the live and dead fungi characteristics of sorption, factors influencing heavy metal removal, and the biosorption capacities for heavy metal ions removal and also discusses the biosorption mechanisms.

## 1. Introduction

Nowadays, contamination of water bodies by heavy metal is becoming a great global concern [1]. The heavy metals reach the environment by two major sources such as natural sources (volcanic emissions, deep-sea vents, forest fires, and geysers) and anthropogenic sources (mining and smelting sites, painting and coating industries, metal-manufacturing plants, and tanneries). Expansion of industries leads to an unmanageable release of heavy metals to the environment. This problem is highly observed particularly in developed countries that produce huge quantities of wastewaters that contain a high concentration of heavy metals [2–5]. These metal ions are persistent and nondegradable in the environment. Accumulation of heavy metals is the result of the disposal of concentrated metal wastes by industries [6]. Due to improper management, these heavy metals generated from different industries as effluent reach the environment (different water bodies, soil, and air) ultimately, causing environmental pollution, which is becoming a threat to humans as well as other living organisms [3]. Even at a low concentration, heavy metals are the potential to create chronic toxicity. Hence, the removal and recovery of heavy metals from industrial effluent streams are highly needed for the protection of the environment.

As a result of the above problems, the great interest in metal-microbe interactions has risen by different scholars and scientists to find suitable methods for tackling and stabilization of heavy metals in waters, soils, and effluents [7]. The problem is observed in both developed and developing countries, but due to lack of technologies, advanced manpower, and low policy enforcements, the challenge is stronger in developing countries. Different conventional methods have been used to remove these contaminants from water bodies including chemical precipitation, filtration, ion exchange, reverse osmosis, evaporation, membrane technology, carbon adsorption, electrowinning, preconcentration, coagulation of wastewater, chelation, redox, and electrochemical treatment [8–13]. Researchers concluded that these technologies have their limitations on cost-effectiveness, complexity, cause of secondary pollution, and alteration of the physical and chemical nature of the environment. Most of these techniques are very expensive for implementation large scale and also dangerous for constant monitoring and control due to their incomplete removal of heavy metals contaminated and unpredictable metal removal [11, 14]. Also, they are impracticable and are not speciﬁc for metal-binding properties [15]. It also removes nontarget useful microbial biota such as nitrogen-fixing bacteria as well as other fauna species [7]. To combat those limitations, biological treatment (bioremediation) methods are highly recommended because they are environmentally friendly, fast, and cost-effective [9, 11, 14, 16]. The other advantages of a biological method can treat a large volume of effluent with low biomass concentration and short operation time [17]. Most known bioremediation techniques include biofilters, biosorption, bioventing, bioaugmentation, biotransformation, composting, land farming, bioreactor, and biostimulation. Biosorption is a biological technique that uses microbes as a biosorbent to detoxify and remove environmental pollutants mainly heavy metals [7]. The microorganism can be involved in heavy metal uptake through two processes, intracellular accumulation through their living biomass, and extracellular binding through both living and dead biomass [4, 16, 18]. Due to their physical and biological nature, fungi have better sorption capacity than the rest of microorganisms [4]. This paper aims to review and compare dead and living fungal biomass methods for heavy metal uptake from polluted environments and examine the factors influencing heavy metal removal as well as the biosorption mechanisms of metal removal by different fungal biomass.

## 2. Source, Effects, and Mechanism of Toxicity of Heavy Metals

Currently, heavy metal contamination is a major global environmental crisis due to its persistency in the environment as a nondegradable matter. In nature, heavy metals are present in forms that are not readily available for uptake by living organisms [15]. Unlike other organic pollutants, heavy metals cannot be broken down by biological or chemical processes. They are essential for living organisms for growth and development under considerable limits [9, 10]. All heavy metals are toxic and cause undesirable effects on organisms, if taken above the standard level. The level of toxicity is depending on the type of living organism, the dose, and the contact time. Most heavy metals are toxic at the concentration level above 10 mg L^−1^; however, mercury (Hg) and cadmium (Cd) can be very toxic at concentrations above 0.001 mg L^−1^ and 0.1 mg L^−1^, respectively [10]. A study by Abdi and Kazemi [19] states that, due to their long persistency, the standard of lead and cadmium in drinking water does not exceed 0.015 and 0.005 mg L^−1^, respectively. They are highly toxic and can be transferred through the food chain via bioaccumulation [1, 10, 20].

The heavy metals responsible for environmental pollution are cadmium (Cd), chromium (Cr), zinc (Zn), nickel (Ni), copper (Cu), lead (Pb), and mercury (Hg) [1, 3, 4, 21, 22] which are generated from different sources. The form and concentration of heavy metal are resolute by the source of contamination. Most industries such as electroplating, metal finishing, metallurgical work, tanning, chemical manufacturing, mining and battery manufacturing, fertilizer, pesticide, and surface finishing [4, 10] generate various heavy metals to the neighboring water bodies and cause severe problems on various living organisms as shown in (Table 1) [3, 19]. Heavy metals such as Hg, Cd, Pb, Zn, Cu, Ni, and Cr are categorized as major water polluting heavy metals [10, 29]. The toxicity of heavy metals is determined not only on human health but also on other life forms starting from disrupting enzyme structures to hair loss. Most heavy metals like lead, mercury, copper, and arsenic can deactivate or inhibit the enzymatic activities of microorganisms [15]. Besides, heavy metal exposure to microbes can change the microbial population size, activity, and diversity, as well as their genetic structure. Apart from microbial effect, exposure to mercury and lead can cause different diseases such as circulatory disorders, joint diseases, nervous system disorders, kidney diseases, rheumatoid arthritis, and damage to the fetal brain in humans and also impaired development, reduced intelligence, and a high risk of cardiovascular disease in children. Cadmium generated from fertilizer and pesticides is able to be a mutagenic, carcinogenic, endocrine disruptor, damage fragile bones, and lungs. Chromium causes human and animal hair loss, headaches, diarrhea, nausea which are the major symptoms of chromium exposure [15].

## 3. Live and Dead Fungi

Fungi are a large and diverse group of eukaryotic microorganisms, three groups of which are of major practical importance including molds, yeasts, and mushrooms [30, 31]. Their cell membrane is composed of a thin, double-layered sheet of lipids, mainly with phospholipids and sterols (approximately 40% of membrane content) and protein molecules (approximately 60%) [32]. From the researchers' perspective, fungal biomasses have a high proportion of cell wall material compared to other biosorption agents, which reveals excellent metal-binding properties. Fungal biomass is utilized for biosorption processes as it often exhibits a considerable tolerance towards metals and other factors, such as low pH [32].

Different scholars carried out several investigations on the removal of heavy metals from a contaminated site using both live and dead fungal biomass because fungal biomass provides a metal sink, either by metal biosorption to biomass or around hyphae [33, 34]. Passive adsorption process on cell surface was performed using inactivated (dead) fungal biomass and active sorption process was also performed using live biomass [35]. Dead biomass was considered to be superior to live ones for various reasons in Table 2.

## 4. Factor Influencing the Removal of Heavy Metals Using Dead and Live Fungi Biomass

### 4.1. The Influence of pH

The most important parameter influencing the sorption capacity is the pH of the adsorption. Removal of Cd was increased by reducing the pH level in live and dead-mode experiments [39]. According to Ezzouhri et al. [40], Pb^2+^ biosorption capacity using *Penicillium* sp. is strongly pH-sensitive and adsorption increased with the increase in pH. Maximum biosorption capacity determined as 60.76 and 52.09 mg g^−1^ was obtained at pH 5.5 for dry and wet biomass, respectively. The highest potential of Cd uptake by *Aspergillus versicolor* was found at a pH of 4 for live and dead biomasses and a pH of 6 for dried biomass. The biosorption of dried biomass increased with the solution pH. At a pH of 6, due to the more amounts of OH ions within the solution, the binding sites on the fungal cell wall are negatively charged [39]. The maximum removal of chromium by dead biomass of *Trichoderma* sp. BSCR02 was (82.3%) observed at pH 5 [41]. The uptake and percentage removal of Cd (II) using *Aspergillus fumigatus* were reported to be 5.21 ± 0.23 mg g^−1^ and 70.32 ± 1.21% and increased up to pH 5.0 and gradually decreased with further increase in pH [42, 43].

According to Liu et al. [44], the maximum Cd and Zn uptake capacities using living *Aspergillus niger* are 15.1 mg g^−1^ and 18.25 mg g^−1^ at pH of 4 and 6, respectively. The maximum removal potential of zinc *Fusarium* spp. is 62.0% for dead biomass and 42.3% for live biomass at pH 6 [45]. Removal of Zn varied with alteration in pH, with live and growing *Aspergillus flavus* RH07 and *Aspergillus fumigatus* RH05. The optimal pH for both strains was different for growth and Zn removal, 5.0 and 4.0, respectively. Biosorption per biomass (dry weight) of *Aspergillus niger* was found to be an inverse function of pH, decreasing with increasing pH [46].  Table 3 shows the optimum parametric condition for biosorption of heavy metals with fungal biomass.

### 4.2. The Influence of Initial Concentration

The initial metal ion concentration in the solution plays a major role as a dynamic force to conquer the mass transfer resistance between the solid and aqueous phases [53]. The amount of adsorbed Pb^2+^ per mass unit increased with an increase in the initial lead ions concentration. The maximum lead uptake capacity of wet and dry cells was determined as 59.47 and 52.2 mg g^−1^ at 100 and 200 mg L^−1^ initial Pb^2+^ ion concentration for dry and wet *Penicillium* sp. biomass, respectively [40]. The uptake and % removal of Cd (II) from the liquid medium by *Aspergillus fumigatus* decreased from 3.82 ± 0.72 mg g^−1^ to 2.74 ± 0.20 mg g^−1^ and 75.8 ± 2.35% to 38.25 ± 1.39%, respectively, with increasing concentration from 100 to 500 mg L^−1^. After attaining the optimum concentration, the efficiency decreased due to an increase in a metal dose which is beyond the toxic threshold to the fungus [42]. The metal ion uptake by *Beauveria bassiana* increased with the increment in the initial ion concentration of heavy metals and maximum metal ion uptake was measured as 12.2 mg g^−1^ for Cu (II), 13.5 mg g^−1^ for Cd (II), 12.2 mg g^−1^ for Zn (II), and 11.3 mg g^−1^ for Cr (VI) at 100 mg L^−1^ initial heavy metal concentration [54, 55].

### 4.3. The Influence of Temperature

The temperature of the adsorption medium is considered to be a significant parameter for the energy-dependent mechanism in biosorbent mediated metal removal. The maximum removal of iron by *Aspergillus versicolor* was found to be 22.2 mg g^−1^ at a temperature equals to 31°C. Temperature affects the cell wall stability components, configuration, and ionization of chemical moieties and energy-independent mechanisms are likely to be affected due to temperature changes since the process responsible for removal is largely dependent on the physiochemical characteristic of the medium [56]. According to Faryal et al. [46], maximum Zn uptake by strains *Aspergillus fumigatus* RH05 and *Aspergillus flavus* RH07 fungal strains was observed at 28°C as the optimal growth temperature. An increase in the temperature led to a reduction of Zn removal. The most optimal temperature for heavy metal removal using *Beauveria* was 84.5%, whereas at 20°C and 40°C, heavy metal removal percentage decreased to 40.4% and 43.0%, respectively [53]. The optimal temperature for removal of Cu (II) by *Aspergillus flavus* was at 26°C with a removal efficiency of 40.8% and *Aspergillus niger* at 37°C for Pb(II) removal with a removal efficiency of 45.5% [57].

### 4.4. The Influence of Contact Time

The rate of metal ion biosorption is rapid in the initial period (within an hour) with nearly 90% of the metal-binding because all the active sites are free and accessible for biosorption. But with the increase in time, the biosorption efficiency decreases due to a rise in the saturation percentage of metal ions remaining in the solution [58]. Rapid biosorption of lead by dead *Rhizopus* sp. and *Aspergillus niger* was observed within 60 min of biosorption capacities that were 9.21 and 8.94 mg Pb (II)/g biomass (92.1% and 89.4% of Pb (II) removal), respectively [59].

Fungal species that include yeast (*Penicillium; Saccharomyces*), molds (*Aspergillus; Rhizopus*), and mushrooms are the most known biosorbent of heavy metals [15, 17, 59]. A filamentous fungus is more effective in metal removal than other fungal species from liquid substrates [12]. The metal uptake potential of various fungi has shown in (Table 3). According to Cai et al. [12] study, among other filamentous fungi, *Aspergillus* spp. were the most resistant to heavy metals copper, cadmium, and nickel. Congeevaram et al. [60] also stated that *Aspergillus* spp. can also be efficient in removing 60% of chromium metal. Fungal species such as *Trichoderma atroviride*, *Trichoderma harzianum*, and *Trichoderma virens* are filamentous fungi used in the uptake of zinc, copper, lead, cadmium, and arsenic [7]. Yeasts are also used in heavy metal removal from aqueous solution due to having extracellular glycoproteins. Several yeast species such as *Saccharomyces cerevisiae, Candida albicans, Pichia anomala, Candida tropicalis,* and *Cunninghamella elegans* emerged as efficient sorbents of heavy metals [8, 38]. *Saccharomyces cerevisiae* can sorb 60% of uranium within the 15 min contact time [15]. The two oyster mushrooms *Pleurotus ostreatus* and *Pleurotus eous* are the most well-known fungus for the removal of heavy metals in aqueous solution as mentioned by different authors. A study done by Suseem and Mary Saral [61] shows that *Pleurotus eous* has high lead uptake efficiency of about 93.2% than Cr (27.6%) and Ni (39.8%). A study done by da Rocha Ferreira et al. [11] concludes that *Pleurotus ostreatus* is also highly significant in the biosorption of heavy metals through its biomass from polluted water. Lead, cadmium, and chromium are the most metals sorbed by *Pleurotus ostreatus* with uptake efficiency of 99.9–100.0%, 45.9–61.1%, and 29.4–64.5%, respectively [62].

## 5. Mechanisms of Heavy Metal Uptake by Dead and Live Fungal Biomass

Different authors define biosorption in various terms. According to Abdi and Kazemi [19] and Sharma et al. [17], it is the passive uptake of metal ions by dead/inactive biomass from an aqueous solution. According to Chatterjee [14], Gadd [4], and Javanbakht et al. [16] studies, biosorption is an activity of both living and dead biomass to uptake metal ions from an aqueous solution. The same study done by Gadd [4] also points out the same result. A fungus has advantages such as an assortment of functional groups due to the nature of the cell wall, ease to grow at a large scale, unsophisticated fermentation techniques, and inexpensive growth media [38] and also is abundantly available as industrial waste products such as waste from organic acid and beverages [28]. The major dry weight of the fungus cell wall is due to the composition of 80% to 90% of polysaccharide, proteins, and lipids such as glucans, chitin, mannans, and phosphomannans [16, 17] These cell wall compositions contain various metal-binding function groups such as hydroxyl, carbonyl, chitin, acetamide, carboxyl, sulfhydryls, thioether, sulfonate, amine, amide, imidazole, phosphonate, and phosphodiesters [12, 15]. These chemical bonds are responsible for providing the ligand atoms to form metal ions complexes by attracting and retaining metals in the biomass. The anion formed by the functional groups enables it to bind with a metal cation [15]. The factors such as the cellular surface of the fungus, the exchange of metal ions, and the formation of metal ions complex play a major role in the sorption of heavy metals in an aqueous solution. In addition to biosorption, the fungus can reduce the toxicity level of heavy metals. Once metals are attached to ligands formed by functional groups on the cell surface, they can convert from one state of oxidation state to the other through different forces of attraction [15].

Two types of fungus biomass are used in the sorption process, live or dead (inactivated) process [12, 18].

The pathways of metal uptake are through multiple processes such as binding to the cell surface, intracellular accumulation, extracellular precipitation, and volatilization [14]. Biosorption mechanism may be classified according to dependence on the cell's metabolism which is called metabolism dependent or according to the location where the metal removed from solution is found which is called nonmetabolism dependent/metabolism independent like extracellular accumulation/precipitation, cell surface sorption/precipitation, and intracellular accumulation [63]. Biosorption by inactivated biomass is a passive adsorption process solely by cell surface binding; in contrast, live biomass sorption is an active process in which both internal and external cellular metabolism such as detoxification, chelation, volatilization, and bioaccumulation occurred [12, 14, 19]. Studies were done by Li et al. [37] and Iram et al. [57] show that the use of dead biomass in heavy metal uptake offers certain advantages over living cells. In contrast, active biomass sorption is better than inactive sorption, because metal removal by inactive fungus biomass only occurs by physicochemical interaction [36].

Biosorption and bioaccumulation involve interactions and concentrations of toxic metals or organic pollutants in the biomass, either living (bioaccumulation) or nonliving (biosorption) [64]. Bioaccumulation is the gradual accumulation of substances in an organism. It happens when an organism absorbs a substance at a faster rate than the catabolism and excretion processes. Biosorption is sorption and complexation of dissolved metals based on the chemical activity of nonliving microbial biomass or by materials derived from biological sources by the means of passive binding from an aqueous solution and bioaccumulation is an active process based on living cells, in which removal of metals require the metabolic activity of living organisms [65]. Table 2 shows that the main difference between the biosorption of heavy metal by live and dead fungal biomass.

Uptake potential varies with various environmental factors such as metal characteristics, the bioavailability of the metal to the biomass, moisture content, the concentration of pollutants, nutrients, electron acceptors, redox potential, pH, oxygen, osmotic pressure, temperature, and water characteristics [15]. The strain isolated from a polluted environment is more efficient since it adapts well to the environmental conditions and tolerates the toxicity of the heavy metals than isolates of nonpolluted environment [15, 66]. The target areas for efficient isolates are polluted soil, industrial effluent, and waste disposal sites. Metal uptake by fungal cell contains a two-step process: (1) a stoichiometric interaction between the metal and functional group in biosorbent cell surface; (2) the second step is gradual metals deposition [28]. At first, the metal ions in the solution are bind with the cell wall of the biosorbents because the cell wall is the primary component that comes in contact with the metal ions. The site where the metal removed from the solution found is categorized as extracellular accumulation/precipitation, intracellular accumulation, and cell surface adsorption/precipitation [16]. The first two sites happened by active biomass biosorption process which is depending on cell metabolism, while the last location happened by the dead cell biosorption process which is independent of cell metabolism rather than depending on the physicochemical interaction between metal ions and chemical bonds from the biosorbent cell.

### 5.1. Metal Uptake by Dead Biomass

**Figure 1:**
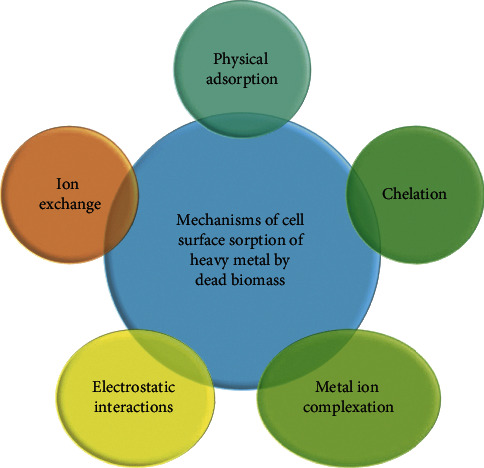
The mechanism of cell surface sorption of heavy metal by dead biomass [17].

It is metabolic independent biosorption activity used through physicochemical interaction between the metal and the functional groups present on the fungi cell surface [16]. According to Dhankhar and Hooda [28], the use of living organisms may not be an effective option for the removal of highly toxic heavy metals due to various factors. Dead biomass has many advantages, including high environmental resistance, greater toxicity tolerance, reasonably fast regeneration and reuse absorbance, high sorbed metal recovery, simple numerical modeling of metal absorption reactors [16], and no need for specific culture media to maintain its active state [11]. The additional benefit of using inactive biomass is no need for nutrition, maintenance, and the biosorbents which can be stored for long periods without any adverse effect on their performance [11, 16]. Metal uptake level of dead cell fungal has been shown to be greater than living cell based on pretreatment methods (a method used to kill the living cell) [18]. The mechanism of cell surface sorption of heavy metal by dead biomass includes physical adsorption, ion exchange, chelation, electrostatic interactions, and metal ion complexation (Figure 1) [17].

The pathway used for surface sorption due to the non-speciﬁc attraction forces such as Van der Waals forces is physical adsorption, which is rapid and reversible. Ion exchange is the substitution of an ion obtained from sorbent when being in contact with another oppositely charged ion from cell wall ligands [4]. The ion exchange method cannot be used on a large scale, because it is extremely expensive, especially when treating a large amount of wastewater containing heavy metal in low concentration [28]. Electrostatic adsorption like physical adsorption is usually rapid and highly reversible and occurs due to the Coulombic attraction forces between sorbent and adsorbent [16]. Some fungi chelate toxic metals and cause the formation of metallo-organic molecules by producing organic acids to make metals more complex [28]. Complexation involves the development of a complex on the cell surface after the interaction between metal-ligand and sorbate-sorbent interactions [16]. The complex consists of one or more positively charged central atoms surrounded by and attached to ligands having usually negatively charged. The ligands are obtained from different functional groups located in the cell wall of fungus including carboxyl, amino, thiol, hydroxyl, phosphate, and hydroxyl carboxyl [11, 14]. This mechanism is mainly used for metals such as copper, zinc, cadmium, and mercury accumulation by *Pseudomonas syringae* [16].

### 5.2. Metal Uptake by Live Biomass

**Figure 2:**
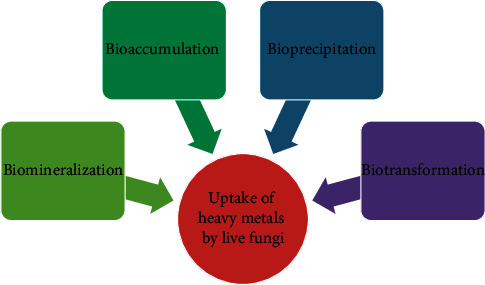
The mechanism of metabolic activity for heavy metal uptake by live biomass [11].

It is an active process whereby uptake of heavy metals requires the metabolic activity of a living organism [13] such as biomineralization, biotransformation, bioprecipitation, and bioaccumulation (Figure 2) [28]. The living biomass cell wall is negatively charged due to legends formed from functional groups easily attached to the metal ions available in the solution. In the case of the second step process, metabolic-dependent intracellular uptake leads to the transportation of metal ions across the cell membrane after cell surface interaction. The pollutant can be transported through the cell membrane to the cell and accumulate intracellular and cell metabolic cycles [28].

## 6. Conclusion

The utilization of fungal biomass to remove heavy metals and/or to recover economically valuable metals from wastewater is attractive in industrial wastewater treatment. The process of heavy metal removal has many attractive features including the removal of metal ion over a relatively broad range of temperature and pH. This is principally due to the characteristic of the fungal cell wall which consists of a significant volume of polysaccharides, proteins, and large functional groups that can interact with heavy metals by diverse chemical forces. Moreover, fungi possess numerous mechanisms (physical adsorption, absorption, precipitation, complex formation, bioaccumulation, biomineralization, and biotransformation) and anionic functional molecules to remove heavy metal ions and hence are viewed as promising biosorbents. However, future studies are recommended to explore the efficiency of fungal biomass for the biosorption of mixed complex pollutants and posttreatment studies of fungal biomass after biosorption. The use of genetically modified strains for specific industrial applications can become one of the main subjects of biosorption engineering in the future to prepare economically attractive analogous sorbent materials.

## Figures and Tables

**Table 1:** Different sources and effects of heavy metals and their mechanism of toxicity.

Metals	Sources	Effects	Mechanisms of heavy metal toxicity	Permissible limits (mg/L)	References
Chromium	Metal plating, electroplating, leather, mining, galvanometry, and dye production	Normocytic, hypochromic anemia, leukopenia, disturbing the vegetable yield and its quality to humans (i) Reduction in root growth, leaf (ii) Inhibition of seed germination (iii) Reduction of protein content in algae and photosynthetic pigments	Reactions between Cr^6+^ and biological reductants like thiols and ascorbate	0.5	[10, 23, 24]

Lead	Industrial sources, mining, plumbing, and fuels	Mental retardation in children, lung, and kidney damage Disturbs various plant physiological processes (i) Fastens the production of reactive oxygen species (ROS) (ii) Lipid membrane damage	An imbalance between the production of free radicals and the generation of antioxidants to detoxify the reactive intermediates (i) Replacing other divalent cations like Ca^2+^, Mg^2+^, and Fe^2+^ and monovalent cations like Na^+^	0.001	[4, 24]

Cadmium	Electroplating, fertilizers, mineral processing, and battery manufacturing	Kidney damage, cancer, gastrointestinal disorder Influencing the enzymatic systems of cells and oxidative stress and inducing a nutritional deficiency in plants	(i) Binding to cysteine-rich protein such as metallothioneins (ii) Binding with cysteine, glutamate, histidine, and aspartate ligands	0.003	[4, 10, 24]

Arsenic	Mining by-product, pesticides, chemical waste, and fossil fuel burning	Internal cancer, skin lesions bronchitis, dermatitis, and death (i) Acute poisoning (ii) Affect the quality of surface water	(i) Biotransformation of harmful inorganic arsenic compounds get methylated by bacteria, algae, fungi, and humans to give monomethylarsonic acid (MMA) and dimethylarsinic acid (DMA)	0.01	[4, 10, 24]

Mercury	Batteries, paper industry, metallurgy industries, chemical manufacturing, and mining, coal	Damage to the central nervous system, protoplasm poisoning, increased heart rate (i) Microtubule destruction, mitochondrial damage, lipid peroxidation, and accumulation of neurotoxic molecules (ii) Malfunctioning of nerves, kidneys, and muscles	(i) Binding to freely available thiols as the stability constants (ii) Attachment to the selenohydryl and sulfhydryl groups	0.001	[4, 6, 10, 24]

Zinc	Refineries, brass manufacture, metal plating, and plumbing	Damage to the nervous membrane, corrosive effect on the skin	(i) Generating reactive oxygen species (ii) Activation of the mitogen-activated protein kinase pathway	5.0	[7, 25]

Manganese	Mining, industrial waste, acid mine drainage, welding, and fuel addition	Damage to the central nervous system	(i) It is added to gasoline as methylcyclopentadienyl manganese tricarbonyl (MMT)	0.04	[4, 7, 26]

Copper	Copper and brass plating, mining, metal industries, and copper-ammonium rayon industries	Liver and kidney damage inducing DNA strand breaks and oxidation of bases via oxygen-free radicals	(i) Reacting with several biomolecules (ii) Participating in the formation of reactive oxygen species (ROS)	1.5 mg/l	[10, 24, 26]

Nickel	Nickel- or chromium-plated taps, bore-hole equipment	Skin sensitizer, dermatitis, and prenatal mortality	(i) Replacing the essential metal of metalloproteins (ii) Binding to catalytic residues of nonmetalloenzymes	0.020	[26, 27]

Cobalt	Aircraft engines, magnets, grinding and cutting tools, artificial hip and knee joints, glass, ceramics, and paints	Congestive heart failure, dermatitis, liver and kidney effects, nausea, vomiting, diarrhea, bleeding, and coma	Generating superoxides Generating free radical		[26, 28]

**Table 2:** Comparison of live and dead fungi sorption features.

S/no.	Sorption characteristics	Sorption by dead biomass	Sorption by living biomass	Reference
1	Cost-effectiveness	Utilize less cost	Utilize high cost	[36]
2	Recovery of toxicant	Possible	Difficult	[36]
3	Regeneration and reuse activities	Possible to reuse various cycle	Difficult	[28, 36]
4	Energy demand	Low energy demand	Energy is highly required	[36]
5	Rate of removal	Rapid	Usually slow due to intercellular accumulation	[36]
6	Selectivity	Poor, but can be improved by modification/processing of biomass	Better	[28, 36]
7	pH	Strongly affect sorption capacity	Partially sorption capacity	[28, 36]
8	Maintenance	Easy	Difficult	[36]
9	Cell disruption	No	Yes	[37, 38]
10	Percentage of heavy metals removal	High	Low	[37, 38]
11	Desorption efficiency	High	Low	[37, 38]
12	Recovery and reuse potential of the cell	High	Low	[37, 38]
13	Binding sites and functional groups	More	Less	[37, 38]
14	Modeling and analysis	Easy	Difficult	[4]

**Table 3:** Comparison of some live and dead fungal biomass for heavy metal removal using optimal experimental conditions.

Fungal species	Heavy metal	Biomass type	Optimum parameters						Adsorption capacity (mg/g)	References
			pH	Initial concentration (mg/L)	Temperature (°C)	Bisorbent dose (mg/L)	Agitation speed (rpm)	Contact time (min)		
*Streptomyces ciscaucasicus*	Zn (II)	Live	5	150	28	2000	90	480	42.75	[37]
		Dead	5	150	28	2000	90	480	54	

*Pleurotus ostreatus*	Cr (VI)	Live	5.6	—	28 + 2	169.84	—	648000	—	[1]
		Dead	5.6	—	28 + 2	368.21	—	22	—	

*Pleurotus ostreatus*	Cd (II)	Dead	6	10	26 ± 1	500	125	10	—	[47]

*Mucor rouxii*	Pb (II)	Live	5	10	—	50	—	420	35.69	[48]
		Dead	6	10	—	50	125	300	53.75	

*Trametes versicolor*	Cd (II)	Live	5.5	600	25	25	400	60	102.3 ± 3.2	[49]
		Dead	5.5	600	25	25	400	60	120.6 ± 3.8	

*Aspergillus fumigatus*	Pb (II)	Live	5	250	28 ± 1	150	150	300	21.579	[50]
		Dead	5	10	28 ± 1	0.04	150	280	3.651	
	Cd (II)	Live	5	250	28 ± 1	—	150	300	6.286	
		Dead	5	10	28 ± 1	0.04	150	280	0.83	

*Rhizopus arrhizus*	Ni (II)	Live	6	100	25	—	150	360	169.84	[51]
		Dead	6	100	25	0.5	150	360	368.21	

*Lentinus edodes*	Hg (II)	Live	6	25–600	15–45	—	—	—	336.3 ± 3.7	[52]
		Dead	6	25–600	15–45	—	—	—	403.0 ± 2.9	
	Cd (II)	Live	6	25–600	15–45	—	—	—	78.6 ± 2.6	
		Dead	6	25–600	15–45	—	—	—	274.3 ± 3.6	
	Zn (II)	Live	6	25–600	15–45	—	—	—	33.7 ± 1.6	
		Dead	6	25–600	15–45	—	—	—	57.1 ± 1.1	

*Aspergillus niger*	Pb (II)	Live	4	—	—	—	—	120	2.25	[34]
		Dead	4	—	—	—	—	120	7.24	
	Cd (II)	Live	4	—	—	—	—	120	1.31	
		Dead	4	—	—	—	—	120	3.43	
	Cu (II)	Live	4	—	—	—	—	144	0.75	
		Dead	4	—	—	—	—	144	2.66	
	Ni (II)	Live	5	—	—	—	—	192	1.75	
		Dead	5	—	—	—	—	192	0.96	

*Cladosporium resinae*	Cd (II)	Live	—	50	28	—	120	2304	—	[14]
		Dead	—	50	28	—	120	672	—	

*Paecilomyces variotii*	Cd (II)	Live	—	50	28	—	120	2304	—	
		Dead	—	50	28	—	120	792	—	

## Data Availability

No data were used to support this study.
